# Transfer kinetics from colloidal drug carriers and liposomes to biomembrane models: DSC studies

**DOI:** 10.4103/0975-7406.76472

**Published:** 2011

**Authors:** Maria Grazia Sarpietro, Francesco Castelli

**Affiliations:** Department of Chemical Sciences, University of Catania, Viale A. Doria 6, 95125 Catania, Italy

**Keywords:** Biomembrane models, delivery system, drug release, DSC

## Abstract

The release of bioactive molecules by different delivery systems has been studied. We have proposed a protocol that takes into account a system that is able to carry out the uptake of a bioactive molecule released during the time, resembling an *in vivo*-like system, and for this reason we have used biomembrane models represented by multi-lamellar and unilamellar vesicles. The bioactive molecule loaded delivery system has been put in contact with the biomembrane model and the release has been evaluated, to consider the effect of the bioactive molecule on the biomembrane model thermotropic behavior, and to compare the results with those obtained when a pure drug interacts with the biomembrane model. The differential scanning calorimetry technique has been employed. Depending on the delivery system used, our research permits to evaluate the effect of different parameters on the bioactive molecule release, such as pH, drug loading degree, delivery system swelling, crosslinking agent, degree of cross-linking, and delivery system side chains.

Biological membranes are constituted by lipid bilayers as their basic structure. Lipid bilayers, which are sheet-like assemblies of amphiphilic lipid molecules held together by hydrophobic interactions between their acyl chains, form the boundaries between intracellular cytoplasm and the cell’s outside environment, as well as between the interior of many of the cellular organelles and the cytoplasm. This lipid bilayer structure was first recognized as the basis for cell membrane architecture in 1925;[1] however, only in 1972, Singer and Nicholson[2] first proposed a fluid mosaic model, to explain the membrane structure.

According to this model, lipids and proteins diffuse freely within the plane of the cell membrane. Since then, large membrane domains (e.g., basal, lateral, and apical membrane regions of glandular, endothelial, and epithelial cells) and lateral microdomain structures (e.g., lipid rafts, caveolae, and coated pits) have been discovered, which reveal the complex nature of the cell membrane structure.[3] Membrane lipids belong to three groups: glycerol-based lipids (phospholipids), ceramide-based sphingolipids, and cholesterol. Phospholipids are further divided into different groups, depending on their hydrophilic head groups: phosphatidylcholine (PC), phosphatidylethanolamine (PE), and phosphatidylserine (which are largely present in the cellular membrane); in addition phosphatidylinositol and cardiolipin are present in smaller quantities. Membrane constituents are not always homogeneously arranged in the bilayer membrane of biological cells, but rather organized in complex lateral microdomains. This polymorphic nature of lipid arrangement, in addition to a significant variety of lipids, with distinctly different physical properties (i.e., cross-sectional area, fluidity, electric charge, molecular weight), make the lipid membranes extremely intricate structures. Furthermore, the covalent association of proteins and carbohydrates adds to the complexity of this membrane’s structure. As a consequence of this intricacy of the cell membrane structure, along with the highly dynamic nature of the lipid–lipid and lipid–protein interactions in the cell membrane, the biophysical interactions with drugs and drug delivery systems are very difficult to investigate. Therefore, simplified artificial membrane systems, mimicking the natural bilayer lipid membrane, have been developed.

In this article, we show how differential scanning calorimetry studies of biophysical interactions with lipid model membrane systems permit to better elucidate the absorption process of a drug by a biological membrane after the release of the drug from a delivery system or after the dissolution process.

## Biomembrane Models

Biomembrane models are systems in which the lipid organization mimics the arrangement of lipids in natural cell membranes. Supported lipid bilayers,[4] lipid monolayers,[5,6] and liposomes[7] are widely used biomembrane models. In this contest just a brief description of liposomes, which are extensively used in our research, will be provided.

Liposomes are spherical lipid vesicles, with internal aqueous compartments. This simple structure still possesses two fundamental properties of the biological membranes: (i) due to the hydrophobic effects, membranes tend to form closed shapes, which are called vesicles; (ii) lipid molecules can move around (diffuse) rather rapidly and freely within the membranes, as they are usually in the fluid state.[8]

The use of a liposome as a tool to determine the ability of biologically active compounds (BA), to interact, dissolve, and penetrate a lipidic bilayer has been studied over the years, and a vast amount of research has been carried out in an attempt to evaluate the ability of liposomes (MLV or LUV), to operate both as a barrier in mimicking biological membranes or as a carrier in the transport of BA. The structural units of liposomes are amphiphilic molecules, especially phospholipids. The most abundant lipids in liposomes are phosphatidylcholines (PCs), in which a glycerol bridge links a hydrophobic part with a hydrophilic polar head group. In contact with water, PCs form bilayers, due to their molecular tubular shape, differently from detergents, which form micelles because of their conical shape. Other polar lipids such as sphingolipids and amphiphiles can be introduced in the liposomal bilayers. Cholesterol or other sterols, isolated from natural sources, and several lipid conjugated polymers, may be found in the liposome bilayers. The phospholipids exhibit thermotropic mesomorphism. When heated, they undergo a number of phase changes before reaching the fusion. Frequently occurring among them are the lamellar subgel L_c_ and the gel L_β_ phases, which are stable at low temperature, and the lamellar liquid crystalline L_α_ phase, stable at higher temperatures. The biological significance of the latter phase is well known, as it is accepted that the cellular membranes are liquid crystalline bilayers with proteins embedded in them.[9]

The thermodynamic properties of hydrated lipids depend on the molecular structure and on the composition of the lipid dispersion. The phosphatidylcholine phase behavior is affected by hydrocarbon chain length, unsaturation, asymmetry, and branching, as well as the type of chain–glycerol linkage and the position of chain attachment to the glycerol backbone, the head group modification, the stereochemical purity, and the morphology of the lipid aggregates (unilamellar and multi-lamellar vesicles). In addition the phase behavior is influenced by the composition of the aqueous dispersing medium.[10]

A lipid thermodynamic database (LIPIDAT) collects, in one central location, all information on lipid mesomorphic and polymorphic transitions and miscibility. The database is considered comprehensive for glycerophospholipids, sphingolipids, glycoglycerolipids, and biological membrane lipid extracts.[11]

This thermotropic behavior is investigated in depth by the calorimetric technique applied to the studies on lipids and biomembranes. A pronounced and easily detectable effect of solutes on lipid phase behavior is the shift, upward or downward, of the transition temperature. However, the other thermodynamic characteristics of the lipid phase transitions, enthalpy, transition width, and maximum specific heat, can be also influenced by solutes.

As the biomembranes are constituted by a multitude of lipids, these studies were mainly carried out on synthetic and a single kind of lipids, so that the interaction between BA and lipids could be easily considered.

It has been several years since our research group is involved in the study of the release of drugs from delivery systems to biomembrane models using the DSC technique. In this article the main results obtained from such studies will be presented. The steps of the protocol used are as follows:

- To evaluate the interaction between the drug under study and the biomembrane models
- To determinate the real amount of drug present in the phospholipid and the aqueous phases of the biomembrane model dispersion
- To evaluate the factors affecting the kinetic of absorption of the free drug by biomembrane models
- To ascertain that the delivery system does not interact with the biomembrane models
- To evaluate the release of the drug from the delivery system to the biomembrane model

In addition a brief description of the DSC technique will be provided

## Differential Scanning Calorimetry

Differential scanning calorimetry (DSC) is the most frequent technique used for determining the thermal effects of a variety of materials, including biological systems that are characterized by an enthalpy change and temperature variation. Besides being involved in the determination of the effect of hydration, pH, solvent, and kind of composition, on the phase transition, and of the changes of enthalpies of model lipid membranes and phospholipid bilayers, it is used in the thermal characterization of complex processes such as the denaturation of proteins and to study glass transition of polymers. Differential scanning calorimetry scans temperature and measures the difference between the heat flows to a sample and a reference pan that is under the same temperature program, at atmospheric pressure, and measures the heat capacity of a material. Differential scanning calorimetry measures the heat flow going into or being released by a material. From that, the heat capacity at constant pressure (*Cp*) can be calculated. Heat capacity units are cal °C^−1^ or J °C^−1^. It measures the amount of heat input (q) required to raise the temperature of a specimen by one degree Celsius while at constant pressure. Heat capacity is usually normalized by dividing the specimen heat capacity by the number of grams, to get the heat required to raise one gram of specimen by one degree Celsius. This corresponds then to the specific heat capacity *Cp*. If desired, the heat capacity can be normalized by the number of moles. Heat capacity is defined by *Cp* = (δ*q* / δ*T*)*p*,where T is the temperature and *q* is the heat input.

If the temperature changes from T_0_ to T_1_, the enthalpy of the reaction $\left(∆H\right)\;\mathrm{is}\;∆H\;=\;\int_{T_{0}}^{T_{1}}CpdT$

Usually, ∆T is small, and *Cp* is independent of temperature between *T*_0_ and *T*_1_ . The integral thus reduces to ∆*H* = *Cp* (*T*_1_ - *T*_0_) = *Cp∆T*.[12]

## Interaction Drug / Biomembrane Model

The first step to be taken into account in the study of the release of a BA from a delivery system to a biomembrane model is the evaluation of the interaction of the BA with the biomembrane model.

In order to evaluate this interaction, the lipid vesicles are usually prepared in the absence and in the presence of increasing amounts of BA, and the measurement of the effect of the BA on the thermotropic behavior of the phospholipid bilayers (T_m_ and ∆H), applying the van’t Hoff model, is carried out.[13] It is, in fact, known that, for dilute solutions, the presence of a solute in the solvent can modify the thermodynamic parameters (such as the melting temperature) of the solvent. The solute acts as an impurity toward the solvent and the modification is dependent on the amount of the solute. In a similar way, the presence of BA in the ordinate lipidic structure can affect the thermodynamic parameters of the transition from the ordinate gel phase to the disordered liquid crystalline phase.[14–17] The effect is correlated to the amount and to the collocation (for similar compounds) of BA in the lipidic structure.

The biomembrane models (MLV or LUV) are generally prepared following the methods reported a little later in the text, depending on the solubility of the BA:

(a) BA soluble in organic solvent: To prepare the MLV, the phospholipid and the BA are separately dissolved in an organic solvent (generally chloroform / methanol, 1 : 1). Aliquots of the phospholipid solution are distributed in glass tubes in order to have the same amount of phospholipid in all the tubes. In the same tubes aliquots of the BA solution are added in order to have an increasing amount of BA. The solvents are removed under a nitrogen stream (at a temperature higher than the transition temperature of the phospholipid),and then, by freeze drying. To the obtained films a known amount of aqueous solvent at a well-defined pH (generally 50 mM Tris, pH 7.4) is added. The samples are vortexed for one minute and heated for one minute at a temperature higher than the transition temperature, thrice, and then left for one hour in a thermostated bath (at a temperature higher than T_m_). The latter step permits two important processes: the homogeneous repartition of the BA between the lipidic and the aqueous phases and the aggregation of eventual small unilamellar vesicles (SUV).

For the LUV preparation, the MLV, with or without the BA, are repetitively (19 times) passed under moderate pressure at a temperature at least 5°C above the T_m_ through polycarbonate membranes (pores diameter 100 nm) in an extruder system (LiposoFastTM Basic, Avestin Inc.).[18,19] The membrane pores are almost cylindrical, and the vesicles (unilamellar or multi-lamellar) that are larger than the mean pore diameter are reduced in size and lamellarity during the passage through the pores, resulting in a final vesicle size that corresponds to the mean size of the pores.[20,21]

(b) BA soluble in aqueous solvent: To obtain the MLV, a phospholipid solution in an organic solvent is prepared. A BA solution in an aqueous solvent is prepared. Aliquots of the phospholipid solution are distributed in glass tubes so as to have the same amount of phospholipid in all the tubes. The solvents are removed under a nitrogen stream (at a temperature higher than the transition temperature of the phospholipid), and later, by freeze drying. To the obtained films, defined amounts of the BA solution are added, in order to have an increasing amount of BA. The samples are vortexed for one minute and heated for one minute, at a temperature higher than the transition temperature, thrice, and then left for one hour in a thermostated bath (at a temperature higher than the T_m_). The LUV is prepared as described herewith.

To determine the real amount of phospholipid present in each sample, the phospholipid phosphorous content is assessed in the preparation by a phosphate assay, after destruction with perchloric acid.[22]

**Figure 1:**
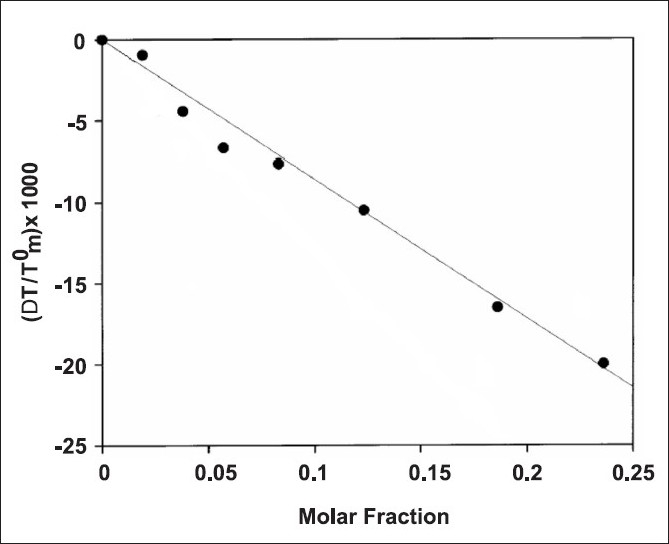
Depression of biomembrane model transition temperature as a function of the molar fraction of BA compound present in the aqueous lipid dispersion. Adapted from Castelli *et al*.[23]

The samples are submitted to DSC analysis and the transition temperature is reported as a variation with respect to the T_m_ of the biomembrane models prepared without BA, as a function of the amount of the BA. Usually, a linear correlation between the transition temperature variation and the amount of BA exists, as shown in Figure 1, where the transition temperature variation is reported as ∆T / T_m_^0^ (∆T = T_m_-T_m_^0^; where T_m_ is the transition temperature of the biomembrane models prepared in the presence of BA and T_m_^0^ is the transition temperature of the biomembrane models prepared without BA). The calorimetric curves obtained from these experiments will be used as reference in the experiments, where the release of a BA from a delivery system to the biomembrane models is studied, as successively described.

## Partition of the Drug between the Lipidic and the Aqueous Phases

When the interaction BA / biomembrane model is evaluated, the repartition of the BA between the phospholipid bilayers and the aqueous medium where the biomembrane models are dispersed have to be taken in consideration.

The obtained graph described earlier [Figure 1] reflects the effect exerted not by the entire amount of BA present in the biomembrane model sample (aqueous lipidic dispersion), but only the effect exerted by the amount of BA present in the phospholipid bilayer. Then, the amount of BA in the phospholipid phase, which really causes the effect has to be determined.

**Figure 2:**
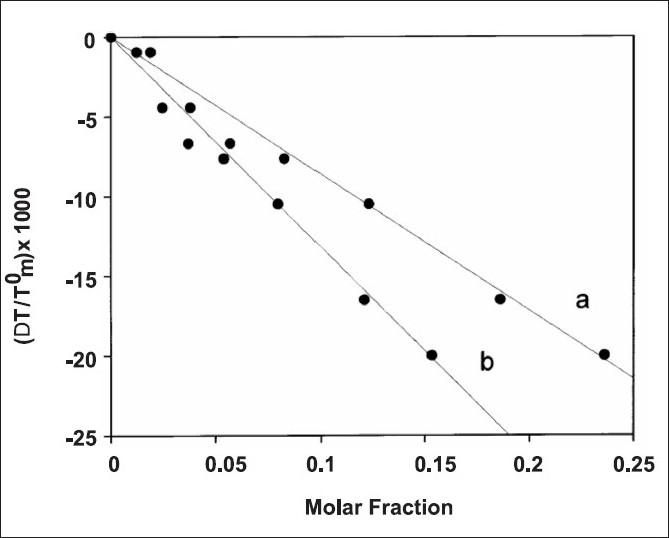
Calibration curve relating the depression of biomembrane model transition temperature to increasing concentration of BA present in: (a) the aqueous lipid dispersion; or (b) effectively dissolved in the lipid matrix. Adapted from Castelli *et al*.[23]

To conduct this, the biomembrane model prepared in the presence of different molar fractions of BA is ultracentrifuged. The supernatant (containing the BA in the aqueous phase) is separated from the pellet (containing the BA in the lipidic phase), whose volume is corrected for the entrapped aqueous volume. The two aliquots are freeze dried and the obtained powders are solubilized in the opportune solvent. The amounts of BA in the two phases are determined by UV / VIS spectrometry. Then, the graph in Figure 1 can be corrected by multiplying the values of the molar fractions (corresponding to the transition temperature variation) for the H_2_O / lipid partition coefficient, and subsequently, the effect on the transition temperature can be attributed to the real amount of BA present in the lipidic phase. In this manner, the obtained line (line b of Figure 2) can be used as the real calibration curve. The values of this curve permit to transform the effect exerted on the transition temperature of the biomembrane model with the amount of BA present in the phospholipid bilayer.[23–25]

In addition, the log P value can give useful information on the lipophilic character of the BA and it can be obtained from the bibliographic data or otherwise it can be assessed by using computational approaches (see for example Pallas 3.0 (CompuDrug International, Inc. San Francisco USA); OSIRIS Property Explorer).[26]

## Transfer of a BA from a Drug Delivery System to a Biomembrane Model

The use of biomembrane models is not limited to getting information on the interaction of BA with the biomembranes. They can, in fact, be used to evaluate the release of drugs from drug delivery systems. Usually, the release kinetic of a drug from a delivery system (hydrogel polymeric systems, nanoparticles, and so on) is evaluated by the use of dissolution systems, which allow to get a graph, where the amount of drug released is plotted as a function of time. This route to evaluate the release does not take into account the absence of a system that is able to take up the drug released during the time, which is instead possible in the presence of a biomembrane model.

To follow the release kinetic with an *in vivo*-like system, the differential scanning calorimetry can be used based on the following protocol.

First of all, from the experiments where the interaction between the BA and the biomembrane model is studied, the molar fraction of the drug that has a strong interaction with the biomembrane model, but at the same time, produces a well-defined calorimetric curve, is chosen as a reference curve for the highest interaction between BA and the biomembrane model. This curve will be considered in all these experiments as the curve toward which that of the biomembrane model should tend if the BA was transferred to the lipid bilayers.

Then, before following the transfer of a BA from a delivery system to a biomembrane model, the release (dissolution) and the interaction of the BA left in contact with the biomembrane model has to be considered. This experiment can clarify the effect, due to the dissolution and the migration and the successive uptake by the biomembrane model in the absence of a delivery system where the BA is molecularly dispersed. The experiments can be summarized as follows:

- An exact amount of the drug is weighted at the bottom of a DSC aluminum pan and a defined amount of biomembrane model dispersion is added
- An exact amount of drug loaded delivery system (containing the drug molar fraction chosen) is weighted at the bottom of a DSC aluminum pan and a defined amount of biomembrane model dispersion is added
- The unloaded delivery system (with the same amount of that at point b) is weighted at the bottom of a DSC aluminum pan and a defined amount of biomembrane model dispersion is added

The pans are sealed and submitted to DSC analysis as follows: (a) A heating scan at the rate of 2°C/minute in a range of temperature starting about 20°C below the transition temperature of the phospholipid employed and ending about 20 C above the transition temperature of the phospholipid employed; (b) Next, the samples are left at this temperature for 60 minutes; (c) A cooling scan to bring the sample back to the starting temperature. These steps are repeated, at least, eight times. By this procedure the interaction of the delivery system and of the drug with the biomembrane model is followed both during the heating step and during the isotherm step, when the phospholipids are in a disordered liquid-crystalline phase. In fact, when the phospholipids are in a disordered phase, the drug is more able to penetrate the multibilayer (MLV) or the bilayer (LUV) and eventually to bind the phospholipids and localize among them. This process is slowed during the cooling step. The degree of interaction of the drug with the phospholipid and then the release from the carrier is quantified through the variation of the biomembrane model transition temperature.

**Figure 3:**
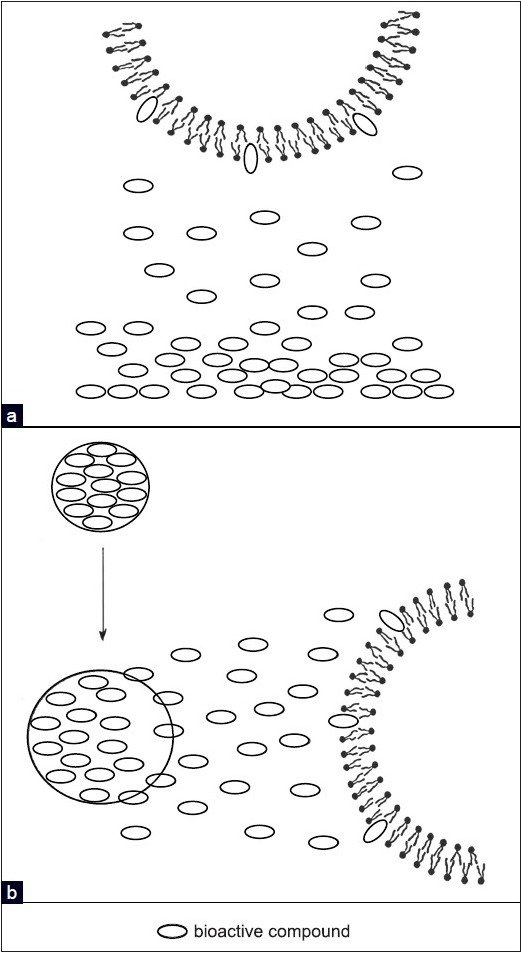
Release of the BA as a free form (a) or from the delivery system (b) and transfer to the biomembrane model

The protocols reported earlier in the text are aimed at obtaining the following information:

- When the free drug is put in contact with biomembrane dispersion, we can not only follow the uptake of the drug by the biomembrane model [Figure 3a], but also the dissolution and diffusion processes through the aqueous phase of the drug, which, in this case, are affected by the uptake process. We can also measure the effect of the pH on the dissolution of the drug and consequently on its interaction with the biomembrane model.
- When the drug loaded delivery system is put in contact with biomembrane model dispersion we can evaluate the uptake of the drug released from the delivery system, the process of hydration of the dissolution of the delivery system, the drug diffusion through the delivery system, and successively through the aqueous phase [Figure 3b]. In this case even the effect of the pH on these processes can be evaluated.
- When the unloaded delivery system is put in contact with the biomembrane model dispersion, we can evaluate the eventual effect of the system on the thermotropic parameters of the biomembrane model. In case we obtain no effects, we can ascertain that when we put the loaded delivery system in contact with the biomembrane model, the effect on the transition temperature is exerted just by the drug. In this process even the effect of the pH can be monitored.

Depending on the use of MLV and LUV, different information can be obtained. Let us consider the use of MLV first.

**Figure 4:**
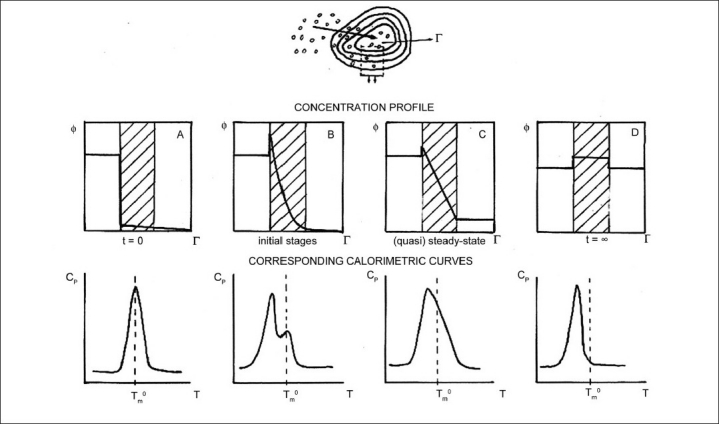
Upper part: the concentration (Ф) profile of a BA inside a multi-lamellar vesicle at different times. *r* is the multi-lamellar vesicle radius. At *t* = 0 all the BA molecules are in the external aqueous medium (A). At intermediate times (B) one observes a nonlinear concentration gradient throughout the membrane which becomes linear in the late stages (steady-state C). At *t* = ∞ the BA concentrations in the external and internal aqueous medium become identical (D). Lower part: the corresponding variation of the specific heat (*Cp*) with temperature (*T*) at different times. *T*_m_^0^ is the melting temperature of the unperturbed multi-lamellar vesicle. Adapted from Raudino *et al*.[27]

The permeation of a multi-lamellar vesicle by a BA, which is in the proximity of the biomembrane model, determines interesting effects on the thermotropic behavior of the membrane. The time variation of the specific heat profile can be used to get information on the BA diffusivity within the multi-lamellar vesicle, as shown in Figure 4. At an early stage of the process the calorimetric curve is similar to that of the pure lipid, with a sharp maximum near the T_m_ of the pure phospholipid; at intermediate times a splitting of the calorimetric peak is observed due to the local inhomogeneities of the composite membrane; finally, in the late stages of the process, the two peaks merge forming a single peak that has shifted to a lower temperature than that of the pure phospholipid phase transition.[27]

Both, when the BA is in a free form or it is contained in the delivery system, three different cases can take place.

- The BA does not transfer or transfers in a very low amount, through the aqueous medium, to the biomembrane model.
- After the first incubation period, the BA transfers through the aqueous medium into the outer MLV layers, and successively, it transfers to the inner layers.
- The BA quickly transfers, partially or completely, to all the MLV layers.

**Figure 5:**
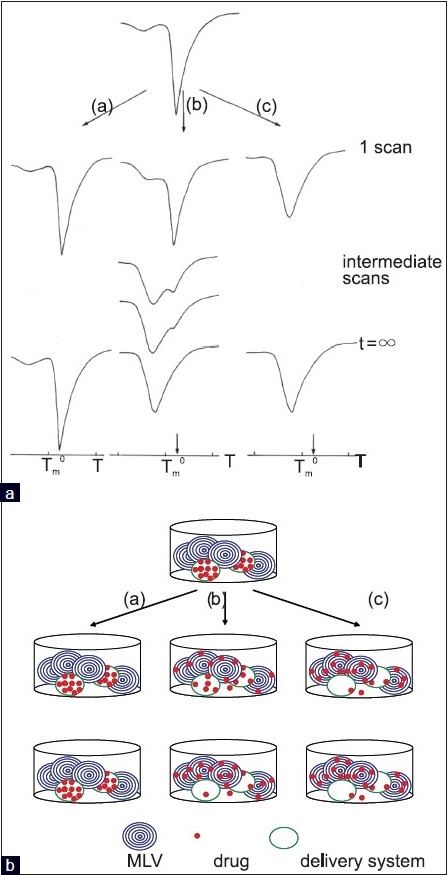
Panel A. Calorimetric curves during the permeation kinetic of a drug released from a delivery system through multi-lamellar vesicles. The arrows mark the position of the multi-lamellar vesicles at t = 0. Adapted from Raudino *et al*.[27] Panel B. Schematic representation of what happens in the calorimetric pan. In (a) the drug is not released from the delivery system and the calorimetric curve remains unchanged. In (b), initially, the drug is released from the delivery system and transferred to the external bilayers of MLV and a double peak is observed in the calorimetric curve; as the time passes the drug localized also in the internal bilayers and a unique peak is observed in the calorimetric curve. In (c) the drug released from the delivery system quickly localizes uniformly in all the bilayers and a unique peak is observed from the first times of contact

Then, the examination of the calorimetric curves permit us to determine which of the three cases occurs [Figure 5]. Let us consider the case (a) the condition of non-transfer of the drug to the MLV is evidenced by the unchanged calorimetric curve during the entire incubation time.

In the case (b), the calorimetric curve associated to the early time of contact between the delivery system and the MLV, is very similar to that of the MLV; as the time passes the main peak splits into two peaks, caused by the inhomogeneous distribution of the BA in the MLV; in particular the BA mostly localizes in the outer bilayers; the successive scans allow the BA to pass into the inner layers by a flip-flop mechanism, in a series of mobile equilibria, among the BA loaded layers, the BA unloaded layers, and the aqueous medium. Such equilibrium brings to the final state and the two peaks merge in an unique peak due to the homogenous distribution of the BA in the multi-lamellar bilayers. The occurrence of case (c) is evidenced by the presence of an unique main peak which, quickly or slowly, moves to a lower temperature than that of the pure phospholipid.

As stated before, by using the MLV we were able to detect the uptake process of a BA (free or delivery system released) by the biomembrane model, by considering the T_m_ shift. If the T_m_ moved toward the value observed when the same amount of BA was placed in the MLV during their preparation, we considered it a complete transfer of the BA into the inner lipid layers.

To be sure that such a process was complete and to better define if the limiting step of the transfer process could be attributed to the dissolution and migration in the aqueous medium, rather than the permeation and partition into the lipid multilayers, LUV were employed.

The LUV, having only one bilayer, do not show the problem of the BA transfer from the aqueous medium to the outer bilayers and then to the inner bilayers; then we can consider, by studying their behavior, the transfer limitation due to the medium surrounding the vesicles.

**Figure 6:**
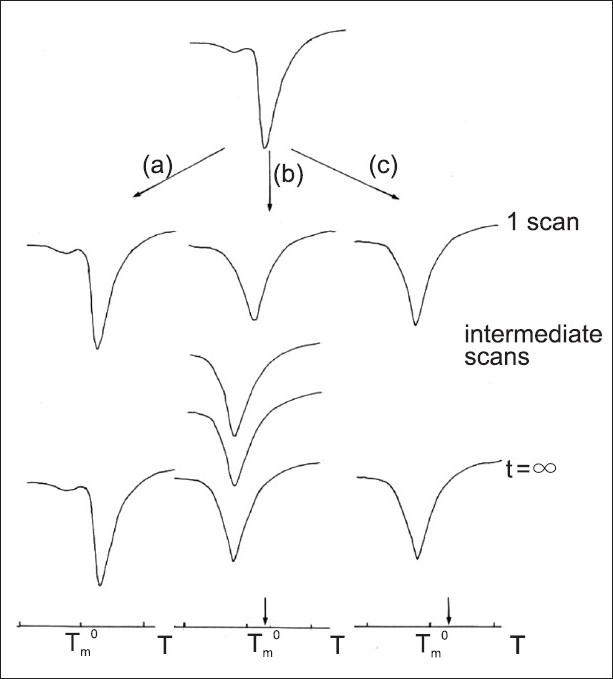
Calorimetric curves during the permeation kinetic of a drug thought unilamellar vesicles. The arrows mark the position of the unilamellar vesicles at t = 0. In (a) the drug is not released from the delivery system and the calorimetric curve remains unchanged. In (b) the drug is slowly released from the delivery system and transferred to LUV and the calorimetric peak slowly moves to lower temperature. In (c) the drug is quickly transferred from the delivery system and transferred to LUV and the calorimetric peak quickly moves to lower temperature. Adapted from Raudino *et al*.[27]

Figure 6 shows the three possibilities that can occur when a BA (free or loaded in a delivery system) is left in contact with the LUV.

As stated before for MLV, in the case (a) the BA is not able to reach the LUV surface even after several incubation periods at a temperature higher than the T_m_ . In this case, processes such as low dissolution rate and unfavorable partition between delivery system and water avoid the BA-LUV interaction.

If the BA dissolves in the medium surrounding it or it is released by the delivery system and migrates to the LUV surface interacting with it, we can differentiate if the limiting step is the process of dissolution or release with respect to the interaction with the LUV bilayer by comparing the curves (b) and (c). In b, it is evidenced that the slow interaction BA-LUV can be ascribed to a lower availability of the drug near the LUV surface; while the curves (c) indicate a fast interaction that can be ascribed to the fast absorption caused by the high availability of the BA.

The interaction observed when a fixed amount of BA is put in contact with an MLV or LUV, compared with that observed when the BA is loaded in the vesicles during their preparation, gives useful information about the processes of dissolution, release, and uptake.

The comparison between the effect exerted by a BA on MLV or LUV (curves (b) and (c) in Figures 5 and 6) allows to determine if the limiting step is the uptake or the transfer process and also gives information about the ability of the BA to cross the lipid layers penetrating inside the MLV. In fact, depending on the rate of interaction, if the transfer of BA from the outer to the inner layers of the MLV is complete, beside a uniform distribution, we will obtain a calorimetric curve similar to that of LUV and to those of MLV, prepared in the presence of the same amount of BA.

This protocol was used for instance to follow the release of model drugs from an inulin-based hydrogel (intended to release the drug in the colon), α,β-polyasparthydrazide (PAHy) hydrogels, α,β-poly(N-hydroxyethyl)-DL-aspartamide (PHEA) hydrogels, Eudragit microparticles, and poly(lactide-co-glycolide) micropheres.

With regard to the release of the drug (diflunisal) from the inulin-based hydrogel, the influence of the drug loading of the hydrogel swelling degree and of the pH on the release was evidenced.[28] In that study, DMPC unilamellar vesicles were used as the biomembrane model. The results obtained at pH 7.4 suggested that with a drug loading of 10.4% (w/w) the hydrogel swelled, but diflunisal, that was not so abundant inside the polymeric network, it would take time to dissolve and migrate through the network. When hydration and swelling occurred, the release would become faster. For the hydrogel with the highest drug loading (24%, w/w) we observed an initial fast release, due to the free drug molecularly dissolved inside the polymer; the following release was also fast, but not complete, perhaps due to the high amount of drug loaded in the hydrogel that could affect the swelling and so the release; or it could be also possible that during dissolution the drug started to crystallize due to the high concentration, forming large crystals that obviously dissolved slowly. The best profile of release was obtained with a drug loading of 17% (w/w); wherein besides a fast initial release, a complete release of the drug occurred, probably due to a good balance between drug dispersion in the polymeric network and swelling of the hydrogel. At pH 4, the diflunisal release from the hydrogel with a drug loading of 10.4% (w/w) was low for all the experimental period, due to the low amount of drug present in the hydrogel, the lower swelling at such a pH, and the low-aqueous solubility of the drug; all these factors contributed to reducing the release of diflunisal. The release of the drug from the hydrogel with a drug loading of 24% (w/w) was slower than that with a drug loading of 17% (w/w), and incomplete, thus indicating that the formation of drug aggregates together with migration through the polymeric network, made it difficult for the dissolution process to occur. Seventeen percent (w/w) represented the best loading, allowing a complete release and transfer of the diflunisal to the biomembrane model.

Dipalmitoylphosphatidylcholine (DPPC) LUV and DSC were employed to study the suitability of the hydrogels obtained by chemical crosslinking of α,β-polyasparthydrazide (PAHy) by glutaraldehyde, as carriers for prolonged release of poorly soluble drugs, and the modulating effects exerted by polymer crosslinking.[23] It emerged that by increasing the polymer crosslinking degree the total amount of transferred drug and the release velocity were decreased. This behavior may be caused by the increase in the number of cruciate bonds in the hydrogels, which cause a free volume reduction, obstructing the passing drug.

Successively, PAHy hydrogels were crosslinked with different agents (ethyleneglycoldiglycidylether (EGDGE) polyvinylalcohol (PVA) and glutaraldehyde (GLU)) and different degree of crosslinking and the release of diflunisal by these hydrogels was carried out with the aim to evaluate the effect of the crosslinking agent and the degree of crosslinking on the release. It appeared evident that the total amount of drug transferred and the release rate were affected by the polymer crosslinking degree (it increased with the crosslinking degree) as well as on the nature of the crosslinking agent.[29]

The study of the ketoprofen release from a derivative of the α, β-polyapartylhydrazide polymer containing hexadecylamine to biomembrane models made of DMPC / DMPA vesicles permitted (i) to demonstrate that the polymer was able to release the drug and (ii) to explain how the release could happen; it was in fact, proposed that the hexadecylamine moiety penetrated into the lipidic bilayers, followed by the delivery of the drug. In this manner the micelles could improve the localized release close to the biological target.[30]

The analysis of the release of ketoprofen from polymeric micelles made of PHEA-C16 and PHEA-PEG2000C16 to DMPC / DMPA vesicles permitted to hypothesize a likely mechanism of drug migration from the micelles to the vesicles: We can suppose that the C16 chains interact with the lipidic bilayer, thus causing the opening of the micellar structure and facilitating drug penetration in the lipidic vesicle.[31]

Biomembrane models made of DPPC MLV were used[32] to study the release of moxifloxacin from uncrosslinked and crosslinked (glutaraldehyde as crosslinking agent) chitosan microspheres intended for pulmonary administration. The results showed that uncrosslinked microspheres swelled rapidly and dissolved, releasing free chitosan that was able to interact with the liposomes (increase of ∆H value), probably altering the biomembrane permeability to the drug. Crosslinked microspheres did not show this property.

The release of diclofenac from Eudragit RS100^®^ microparticles and the effect of the pH on such a release was also investigated.[33] In such a study of DMPC MLV (as biomembrane models), microparticles containing two different amounts of diclofenac (14.26 and 25.0%) and two different pHs (7.4 and 4.0) were used. At pH 7.4, the release process for the microparticles loaded with a higher amount of drug appeared to be faster with respect to the lower loaded microparticles, both being slower than the free drug. In fact, the drug dissolved easily in the external pH 7.4 medium and was readily absorbed by the biomembrane model. Drug release from the microparticles was hindered by the acidic pH, which prevented its dissolution and migration through the aqueous medium to reach the model membrane. The results obtained from this study suggested that the process of drug dissolution through a polymeric matrix could be affected by the amount of drug loaded, but mainly by the pH of the dissolution medium.

The release of flurbiprofen, an acidic drug, from Eudragit RS100^®^ and Eudragit RL100^®^ nanosuspensions, was studied at pH 7.4 and the results obtained from DSC, where a biomembrane model (DMPC MLV) was used, and the dialysis experiments were compared.[34] The results showed a plateau in the dissolution profile of flurbiprofen from the nanoparticles, which was related to an equilibrium among the drug release, the drug ionization in the dissolution medium, and the saturation of the binding sites on the surface of polymer particles. This behavior was ascribed to the fact that the dissolved drug that got ionized in the neutral dissolution medium was readsorbed onto the polymer particles because of the presence of opposite electrical charges. In the dialysis experiments the driving force leading to flurbiprofen release from the nanoparticles was the volume and light alkaline pH of the dissolution buffer; the absence of an uptaking system in the external medium made the release profile more strictly dependent on the nature of the polymers. In fact the two polymers showed similar time-release curves, but with a higher amount of drug released from the more permeable RL matrix. Although, when the biomembrane model was used, the drug release profile was conditioned by the MLV bilayers to capture and retain the drug molecules after their release from the polymeric system. In these experiments the volume of the dissolution medium was much smaller with respect to that of the dialysis experiments. The equilibrium among the drug bound to the nanoparticles surface, the fraction dissolved in the medium, and the amount captured by MLV were strongly affected by the affinity of the drug for the RS or RL polymers. As a consequence, the behavior observed for RL and RS nanosuspensions was quite different. In fact, although the maximum released amount was higher for the RL system due to its higher water permeability, the MLV incubated with RS nanoparticles showed a quicker uptake of the dissolved drug. The comparison of the results obtained from the two different kinds of experiments permitted us to conclude that although in the dialysis tests the permeability of RS and RL polymers was the limiting step of the release of an acidic drug such as flurbiprofen, in the smaller space of the DSC pan, the affinity equilibrium for a nanoparticle surface or MLV bilayers played a big role in determining the overall drug release profile.

Similar results were obtained in a study where the release of ibuprofen from Eudragit RS100^®^ and Eudragit RL100^®^ nanosuspensions was studied.[35]

## Transfer of a BA from a Lipophilic Drug Delivery System to Biomembrane Models

The previous model can also represent a useful approach in studying the transfer of a drug from a lipophilic carrier to the biomembrane model, taking advantage of the use of a drug-loaded MLV, using the following protocol: In the calorimetric pan, equimolar amounts MLV, loaded with a known molar fraction (X = n) of the BA, which mimics a lipophilic carrier, and of the drug-unloaded MLV (X = 0), which mimics the biomembrane model, are put in contact and submitted to calorimetric analysis, in which a heating scan is followed by an isothermal period of one hour at a temperature higher than that of the phospholipid and then by a cooling scan, several times.

**Figure 7:**
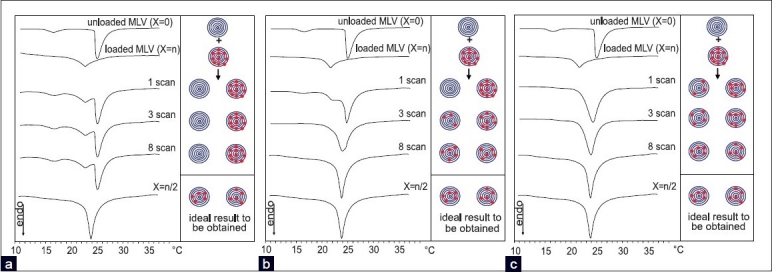
Left part. Calorimetric curves of unloaded MLV (X=0) (which represent the biomembrane model) put in contact with an equimolar amount of loaded MLV (X=n) (which represent the lipophilic carrier), at increasing time of incubation. Right part. Schematic representation of what happens in the calorimetric pan. The calorimetric curves identified as X=n/2 is used as reference and it should be obtained in the case the drug transfers from loaded to unloaded MLV till to have the same concentration in all the MLV which has a mean value among the concentrations of the MLV which were put in contact. (a) The drug does not transfer from loaded MLV to unloaded MLV and the reference curve is never reached; (b) At the beginning the drug does not transfer from loaded to unloaded MLV; as the incubation time passes the drug transfers till its concentration will be the same if all the MLV and the reference curve is reached. (c) The drug quickly transfers from loaded to unloaded MLV and the reference curve is reached

Three different cases can occur: (a) the drug does not transfer from the loaded to the unloaded MLV, (b) the drug slowly transfers from the loaded to the unloaded MLV, and (c) the drug quickly transfers from the loaded to the unloaded MLV. The analysis of the calorimetric curves and their comparison with the calorimetric curve (reference curve) obtained from the MLV prepared in the presence of BA at X = n/2 gives us information on which of the cases occurs [Figure 7]. The curve X = n/2 should be obtained if 50% of BA is contained in the loaded MLV transferred to the unloaded MLV.

If case (a) occurs the calorimetric traces are characterized by two peaks, simply resulting from the sum related to the unloaded and loaded MLV, which were put in contact. This curve remains unaltered for all the calorimetric scans without reaching the reference curve (shape or position).

If event (b) takes place, the calorimetric curves exhibit two peaks related to the unloaded and loaded MLV that were put in contact, in which, as the incubation time passes, the drug transfers from the loaded to the unloaded MLV and the two peaks approach each other (the peak related to the loaded MLV loses the BA and moves toward a higher temperature; whereas, the peak related to the unloaded MLV is enriched with BA and moves toward a lower temperature) and merge in a unique peak, which overlaps the reference curve.

The case (c) is characterized by a single calorimetric peak, due to the fast transfer of the BA from the loaded to the unloaded MLV and with a fast transfer from the outer to the inner bilayers, which gradually moves toward lower temperature. If the transfer is complete the calorimetric curve reaches the reference curve.

This protocol was used with gemcitabine and acyclovir prodrugs obtained by the conjugation of the drug with Squalene.[36,37] These prodrugs were obtained with the aim of increasing the lipophilic character of the drug. It was found that the affinity of the prodrugs to the biomembrane models was much stronger with respect to the free drug and the prodrugs were released from the loaded MLV to the unloaded MLV very gradually, suggesting that the liposomes could be used as a carrier for the sustained release of the prodrugs.

In a recent article of our research group it was shown that trimethylresveratrol transfers slowly from the lipophilic carrier to the biomembrane models; whereas resveratrol exhibits a quicker kinetics of transfer.[38] These behaviors can be explained by the different lipophilicity of the two compounds. Trimethylresveratrol being more lipophilic than resveratrol has a bigger affinity for the lipophilic environment, with respect to resveratrol; hence, it leaves the lipophilic carrier slower than resveratrol. In a recent research[39] the transfer of omega-3 fatty acids from a lipophilic carrier to biomembrane model was studied. From that study it has emerged that docosapentaenoic acid (DPA) and docosahexaenoic acid (DHA) possessing the same carbon atoms, but differing in the unsaturation (DPA with five double bonds and DHA with six double bonds) show different behaviors. In fact, the transfer from the loaded to the unloaded MLV is slow and not complete for DPA, but quick and complete for DHA. The smaller and slower transfer of DPA was attributed to its stronger affinity for the lipophilic environment of MLV.

## Toward Modelization of the Combined Release-uptake Kinetics

The great deal of experimental study described in the previous sections can be exploited in order to gain quantitative information about a number of useful parameters related to the release-uptake process in biological systems. This goal can be accomplished by developing a non-trivial mathematical modelization of the involved coupled processes. Here we sketch the minimal physical effects to be considered in a minimal but realistic picture. The starting point for any release kinetics is the transport equation

$$\frac{\partial C_{i}}{\partial t}\;=\nabla J_{i}\left(C_{i}\right)+F\left(C_{i}\right)$$

which states that the time variation of the concentration *C_i_* of a generic i-th species depends upon the difference between the ingoing and outgoing flux *J_i_(C_i_)* inside a generic volume element of the system (the term ∇*J_i_(C_i_)*, the symbol ∇ meaning spatial difference) augmented by a term that describes the concentration variation related to eventual chemical reactions (the term *F(C_i_)*. The flux J_i_(C_i_) can originate from different causes. In the simplest case it originates from the concentration gradients (Fick’s law: *J_i_(C_i_)* ≈ *D_i_gradC_i_*, where *D_i_* is the diffusion coefficient of the i-th species). Things are generally more intricate because the diffusion coefficient *D_i_* may depend on the swelling degree of the carrier’s matrix (see, e.g., Grassi *et al*,[40]), moreover, other complex effects, such as hydrodynamic effects, may contribute.

A similar equation must be used to deal with the uptake of a diffusing molecule. There is, however, a key difference, because a key contribution comes from the ‘immobilization’ of the processes occurring when the diffusant is trapped at a generic binding site. These immobilization effects can be described in a variety of ways, one of the simplest ones is to introduce a fictious chemical reaction, which destroys the diffusing particles when they hit the binding sites within the target. Such an effect modifies the local concentration of the diffusant, and therefore, the whole diffusant motion. In the case of a prescribed distribution of irreversible traps, the kinetic term to be added to the diffusion equation takes the simple form: *F(Ci)* ≈ *k* *(C*($\vec{r}$)– *C_i_)C_i_*, where *C*($\vec{r}$)is the spatial distribution of the target’s binding sites and *k* is the kinetic constant of the binding process (therefore the quantity *C*($\vec{r}$)– *C_i_* describes the number of unoccupied binding sites). The above-mentioned model leads to two coupled diffusion-type equations (associated with the release and uptake processes, respectively) that can be easily solved.

Once the time-varying local concentration of the diffusant has been calculated, we can easily calculate the variation of the calorimetric properties in each region of the sample. Indeed, as previously stated, the transition temperature and the associated enthalpy of a system (e.g., a multi-lamellar liposome) depend on the local concentration of a foreign compound; such a relationship is empirically determined by calibration curves. Thus the real calorimetric signal calculated at different times is proportional to the space concentration of the diffusant averaged over the whole sample.

The advantage of the above sketched mathematical model is twofold:

- It enables one to numerically derive useful parameters such as the diffusion mobility of a drug in different matrices (e.g., a multi-lamellar liposome), the water-lipid partition coefficient, the release kinetic constant from a dissolving matrix, and so on.
- To test the validity of release-uptake mechanisms by the fitting of theoretical and experimental curves.

Studies along these lines will be discussed in a near future.

In conclusion, the use of biomembrane models and differential scanning calorimetry are useful approaches to study the release of a BA from a delivery system, as also the uptake of the BA from biomembranes models. In our researchers we have studied the release of BA from several kinds of delivery systems and we have obtained useful information, not only on the kinetics of the release, but also on the uptake of the BA by the biomembrane. The described approach can be applied to study the BA release from delivery systems other than those described in this article. For example, in some of our studies we have employed the differential scanning calorimetry to characterize solid lipid nanoparticle (SLN) and nanostructured lipid particle (NLP) colloidal carriers,[41,42] developed as alternative systems to the existing traditional carriers (emulsions, liposomes, and polymeric nanoparticles), especially for the delivery of lipophilic compounds and recently, due to its versatility, we have applied the described approach to follow the release of drugs from SLN and NLP, and their uptake by biomembrane models.
